# Weathering the Storm: Harnessing the Resolution of Inflammation to Limit COVID-19 Pathogenesis

**DOI:** 10.3389/fimmu.2022.863449

**Published:** 2022-05-09

**Authors:** Esther Silberberg, János G. Filep, Amiram Ariel

**Affiliations:** ^1^ Department of Biology and Human Biology, University of Haifa, Haifa, Israel; ^2^ Department of Pathology and Cell Biology, University of Montreal, Montreal, QC, Canada; ^3^ Research Center, Maisonneuve-Rosemont Hospital, Montreal, QC, Canada

**Keywords:** resolution of inflammation, macrophage reprogramming, apoptosis, efferocytosis, COVID-19, IFN-β

## Abstract

The resolution of inflammation is a temporally and spatially coordinated process that in its innate manifestations, primarily involves neutrophils and macrophages. The shutdown of infection or injury-induced acute inflammation requires termination of neutrophil accumulation within the affected sites, neutrophil demise, and clearance by phagocytes (efferocytosis), such as tissue-resident and monocyte-derived macrophages. This must be followed by macrophage reprogramming from the inflammatory to reparative and consequently resolution-promoting phenotypes and the production of resolution-promoting lipid and protein mediators that limit responses in various cell types and promote tissue repair and return to homeostatic architecture and function. Recent studies suggest that these events, and macrophage reprogramming to pro-resolving phenotypes in particular, are not only important in the acute setting, but might be paramount in limiting chronic inflammation, autoimmunity, and various uncontrolled cytokine-driven pathologies. The SARS-CoV-2 (COVID-19) pandemic has caused a worldwide health and economic crisis. Severe COVID-19 cases that lead to high morbidity are tightly associated with an exuberant cytokine storm that seems to trigger shock-like pathologies, leading to vascular and multiorgan failures. In other cases, the cytokine storm can lead to diffuse alveolar damage that results in acute respiratory distress syndrome (ARDS) and lung failure. Here, we address recent advances on effectors in the resolution of inflammation and discuss how pro-resolution mechanisms with particular emphasis on macrophage reprogramming, might be harnessed to limit the universal COVID-19 health threat.

## Introduction

Macrophages are a major immune cell type that, since their unveiling in the 1880s by Eli Metchnikoff, were found to execute phagocytosis and be key effector cells in combating foreign invaders as well as in regulating homeostatic functions (1). Although phagocytosis is associated mainly with the containment and eradication of invading pathogens, it also serves constant housekeeping functions. Macrophages are myeloid-derived immune cells involved in regulating both the humoral and cellular immune responses. Moreover, they are also responsible for the clearance of approximately 2 × 10^11^ erythrocytes every day, a crucial contribution to red blood cell homeostasis without which the host would not survive. Macrophages also clear cellular debris, thereby serving as crucial homeostatic “janitors” (2). Notably, the clearance of cellular debris under homeostatic conditions, and particularly during the resolution of inflammation, leads to significant phenotypic and functional changes in these cells; a phenomenon which results in their reprogramming to new fates and tasks. This review will focus on recent advances in the understanding of macrophage function and the effector molecules they produce during the resolution of inflammation, and will discuss how this information can be harnessed for potential treatment of various aspects of COVID-19.

## Macrophage Phenotypes in Inflammation and Its Resolution

### Macrophage Ontogenesis and Differentiation

While it was originally believed that mammal macrophages are derived from bone marrow-originating circulating monocytes, it is now well-accepted that two distinct macrophage origins exist (3). The first form of ontogenesis takes place prenatally, of which the resulting mesoderm-derived macrophages reside in tissues and self-maintain locally *via* longevity or limited self-renewal, independent from definitive hematopoiesis (4). Examples of these yolk-sac-derived primitive macrophages are microglia (in the brain) (5), Kupffer cells (liver) (6, 7), as well as peritoneal and alveolar macrophages (6, 8). These embryonic macrophages constitute the majority of the human macrophage population and persist quantitatively into adulthood (3, 9).

The second form of macrophage ontogenesis involves tissue-infiltrating monocytes of bone marrow origin, which differentiate into adult macrophages. This type of macrophage is the focus of this review, for these monocyte-derived-adult-macrophages are the ones involved, for the most part, in pathology, inflammation, and restoration of homeostasis (10–13).

It is important to note the interplay of tissue resident and monocyte-derived macrophages. For although self-maintaining, resident macrophages are known to readily adapt to their tissue of residence and become essential for their respective organ homeostasis in addition to operating as quiescent sentinels ready to mount an immune response (14, 15). Resident macrophages have also been found to be joined and sometimes replaced by recruited monocyte-derived macrophages under inflammatory conditions (14, 15). An example of this interplay is the microglia, the resident macrophage within the central nervous system (CNS), which, once activated, also recruit peripheral immune cells to the brain (14). Thus macrophages regulate both innate as well as adaptive components of immunity, both in the steady-state form of microglia regulation of CNS homeostasis through removal of damaged or unnecessary neurons and synapses, as well as recruiting CNS-infiltrating macrophages in response to inflammatory or pathological insults (16–18).

Monocytes play an active role in innate immunity as they differentiate into either macrophages or dendritic cells (DCs), two phagocytosing cell types that share certain functions, such as antigen presentation, yet differ in their specialization. However, in addition to being a mere progenitor to professional antigen-presenting cells (APCs), monocytes were found to phagocytose and present antigens on their own (19). Moreover, neutrophils or B cells transfer particulate antigens to monocytes in the bone marrow, a sampling that consequently initiates their differentiation into DCs (20). These mature DCs possess the ability to “remember” the antigen that had been phagocytosed by their progenitors because these monocytes exhibit a less proteolytically destructive nature than professional APCs which results in intracellularly retained antigen peptides. As professional APCs, DCs preserve a relative proteolytic inefficiency and retain phagocytosed antigen for at least two days, whereas macrophages degrade peptides rapidly, a phenomenon which can interfere with T cell priming (20). The efficiency of macrophage antigen degradation can be attributed to lysosomal cathepsins, the main enzymes responsible for intracellular protein degradation (21). Monocyte progenitors of macrophages also contain cathepsin, albeit in the endosomal compartment, potentially explaining their decreased proteolytic activity relative to mature macrophages (20). Although APC differentiation is regulated by monocyte cathepsin expression and localization, the process of monocyte differentiation into their terminal phenotypes is ultimately dependent on the microenvironment to which they are recruited. Thus, signals from cytokines and other effector molecules would ultimately drive their differentiation into macrophages or DCs (22). An additional factor that has the potential to affect the phenotype of the mature macrophage after initial differentiation has been postulated to be the stage of maturation at which the monocyte is recruited to the tissue from the bloodstream (2). Monocytes undergo maturation in the blood, so the time interval they spend circulating before migrating into tissues may define their function. In mice, distinct monocyte populations, termed ‘inflammatory’ and ‘resident’ monocytes, have been identified based on their expression of cell surface markers. Thus, inflammatory monocytes are defined as CX_3_CR1^lo^CCR2^+^Gr1^+^, whereas resident monocytes are defined as CX_3_CR1^hi^CCR2^−^Gr1^−^ (analogous to the human CD14^+^CD16^−^ and CD14^lo^CD16^+^ monocyte populations, respectively, which share phenotypes and homing behaviors with that of their murine counterparts) (2, 23, 24). The expression of these markers and subsequent categorization of murine monocyte populations is primarily based on whether the monocytes exit the blood stream quickly or spend more time in the circulation following their release from the bone marrow. The former subset is recruited to inflamed tissues and the latter to non-inflamed tissues in a CX_3_CR1-dependent manner (23).

Macrophage plasticity does not end with ontogenetic differentiation. Throughout their lifespan, macrophages both release and respond to multiple signals, which shape the heterogeneity of their functions and phenotypes. Recent data suggest that in addition to external signals and cues, the density of a particular macrophage subpopulation regulates its own continued collective activation (25). This process, termed Quorum Licensing, proposes a heterogeneous immune cell activation role for intercellular communication that occurs in primary macrophages and mediates a nonlinear relationship between a potent activating stimulus and population-level cytokine production (25). Single-cell tracking and dynamic modeling indicated the differentiation of only a subset of cells into a particular phenotype based on the population’s experience of cell density, a mechanism which can potentially “amplify local responses to threats and prevent false alarms” (25).

### The M1-M2 Paradigm

**Figure 1:**
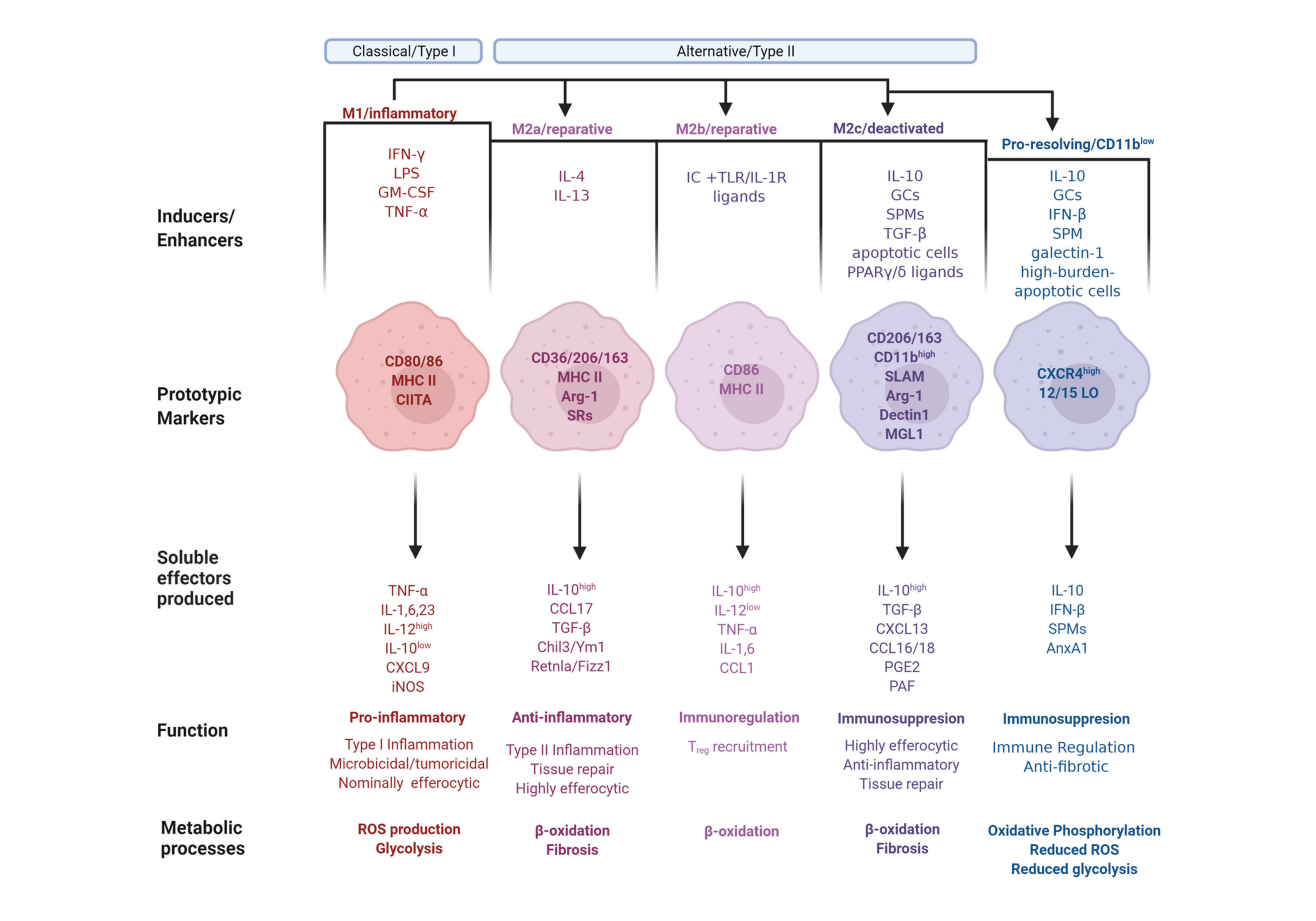
The phenotype continuum of monocyte-derived macrophages. Monocytes migrate into inflamed tissues and differentiate into macrophages that undergo polarization and acquire distinct functional properties in response to environmental signals. Classically activated or type I macrophages (M1) are polarized and generated after exposure to GM-CSF or IFN-γ along with microbial components, such as LPS, or TNF-α. M1-polarized macrophages express the prototypic markers CD80 and CD86, as well as MHC II along with its transactivator, CIITA. M1 macrophages produce pro-inflammatory mediators, including TNF-α, Interleukins (IL) 1, IL-6, and IL-23, as well as CXCL9. The macrophage phenotype dichotomy is underscored by their iNOS/arginase 1 and IL-12/IL-10 balance. Consequently, M1 express high levels of iNOS and IL-12 and low levels of arginase (Arg) 1 and IL-10. M1 polarization results in Type I inflammation, characterized by heightened microbicidal and tumoricidal activities, which are associated with a low efferocytic rate, ROS production and glycolysis. Conversely, alternatively activated or type II macrophages (M2 phenotype) are generally associated with immunoregulatory and tumor-promoting functions. M2 polarization is subdivided into M2a, M2b, and M2c with nuanced immunoregulatory characteristics. The M2a polarization is induced by IL-4 and IL-13, and characterized by expressesion of CD36, CD206 and CD163, MHC II, Arg-1 and scavenger receptors (SR). M2a macrophages produce anti-inflammatory mediators, including high amounts of IL-10, CCL17, TGF-β, Chil3/Ym1 and Retnla/Fizz1. M2a polarization results in Type II inflammation and tissue repair, and is associated with β-oxidation, a high efferocytic rate, and fibrosis. Immune complexes in concert with TLR or IL-1R agonist induce M2b polarization with the markers CD86 and MHC II. M2b macrophages produce immunoregulatory mediators, including IL-1 and IL-6, high amounts of IL-10, and low amounts of IL-12, TNF-α and CCL1. M2b macrophages are involved in the recruitment of T_reg_ cells and undergo β-oxidation. The M2c polarization is induced by IL-10, glucocorticoids (GCs), specialized pro-resolving lipid mediators (SPMs), TGF-β, apoptotic cells or PPARγ/δ ligands, leading to deactivation programs. M2c macrophage markers are CD206, CD163 and high levels of CD11b, as well as SLAM, Arg-1, dectin1 and macrophage galactose-type lectin (MGL) 1. M2c macrophages produce immunoregulatory mediators, including high levels of IL-10, TGF-β, CXCL13, CCL16 and 18, PGE_2_ and platelet activating factor (PAF). M2c macrophages exhibit high efferocytic and anti-inflammatory activities and are involved in tissue repair and fibrosis and undergo β-oxidation. Pro-resolving macrophages are a population, which while displaying M1 or M2 properties underwent an additional phenotype conversion to the CD11b^low^ phenotype. This polarization is induced by exposure to IL-10, GCs, IFN-β, SPMs, galectin-1, or uptake of high-burden apoptotic cells. Pro-resolving macrophages express high levels of CXCR4 and 12/15-lipoxygenase (LO) and release IL-10, IFN-β, SPMs and AnxA1. This macrophage subset is immunoregulatory and anti-fibrotic, and exhibit oxidative phosphorylation, reduced levels of ROS production and glycolysis.

A simplified model of the full spectrum of macrophage activation suggests that functional macrophage phenotypes can be categorized into three distinct groups based on their respective physiological functions: classically activated, alternatively activated or wound healing, and regulatory macrophages (2). Mills et al. proposed a supplementary categorization based on investigations into factors that regulate macrophage arginine metabolism (26), the M1 and M2 subsets. M1 and M2 macrophages express distinct metabolic programs and consequently differ in their abilities to be activated, as well as in their respective qualitative responses to the same stimuli, resulting in opposite influences on inflammatory reactions (26). The M1 and M2 classification, after much subsequent research and elaboration, denotes the bookends of a conceptual continuum on which most macrophage phenotypes fall (26, 27). This spectrum includes all macrophage functional states between pro-inflammation and pro-resolution, or M1 and M2, respectively. The M2 phenotype undergoes additional field conversions into specialized functions, resulting in M2a, M2b and M2c subpopulations (27). Furthermore, in response to the ambiguity that unavoidably arises from the multiple categorizations of macrophage subpopulations and corresponding nomenclature, an additional approach has been proposed, which adduces the basic manipulators of macrophage differentiation; namely post-differentiation stimulation by effectors, like the cytokines IFN-γ or IL-4 (28). Notwithstanding potential equivocacy, we offer here an overview of expanded macrophage phenotype definitions to account for the idea that macrophage activation exists over a spectrum and cannot be bottled into compact categories (**Figure 1**).

Classically activated, or M1 macrophages, produced during cell-mediated, or Type I, immune responses, possess enhanced microbicidal and tumoricidal capacities (2). In response to the characteristic activating stimuli IFN-γ and LPS, M1 macrophages secrete high levels of pro-inflammatory cytokines and mediators, such as inflammatory CC and IFN-γ-responsive chemokines, which drive recruitment of Th1, Tc1 and NK cells to infected tissues (27, 29). Alternatively activated, or M2a macrophages develop after exposure to type II cytokines IL-4 or IL-13, and generate chemokine agonists for CCR3, CCR4 and CCR8, which consequently recruit eosinophils, basophils and Th2 cells (29). M2a macrophages also secrete growth and angiogenic factors, as well as components of the extracellular matrix, thereby enhancing wound healing (2, 30). These macrophages are less efficient than classically activated macrophages at killing intracellular pathogens *via* the production of toxic radicals and pro-inflammatory cytokines. The third category, regulatory macrophages (which is further divided into reparative and pro-resolving subpopulations), are responsible for immunosuppression or the resolution of acute inflammatory episodes. Though these macrophages do have inflammatory capacity, upon encounter with immunomodulatory agents their anti-inflammatory activity is induced. Parasitic, bacterial and viral pathogens appear to exploit this macrophage population to weaken host defense (2). Reparative, or M2b macrophage polarization is dependent on the recognition of immune complexes in combination with stimulation by TLR or IL-1R agonists (27). M2b macrophages are characterized by selective production of CCL1 and consequent recruitment of T_regs_ (29). Their unique cytokine profile indicates that these cells are not anti-inflammatory *per se*, a notion supported by observations of both their protective and pathogenic roles in various diseases (31). The other regulatory macrophage subpopulation is M2c, otherwise known as deactivating, suppressive, or pro-resolving macrophages. Polarization toward M2c is elicited by IL-10, TGF-β, apoptotic cells or glucocorticoids (27). M2c macrophages produce CCL16 and CCL18, which consequently promote recruitment of eosinophils and naive T cells, respectively (29). M2c macrophages are associated with deactivation programs, such as downregulation of pro-inflammatory cytokine production, increased debris scavenging activity, and the initiation of pro-healing functions (27). The distinct macrophage properties during their differentiation are depicted in **Figure 1**.

The transition of macrophages through this continuum is confined to and differs between tissues, a critical quality that distinguishes macrophages from dendritic cells. Although also defined as being immune sentinels, dendritic cells translocate to draining lymph nodes to activate T cells and trigger adaptive immunity (32). Macrophages are the chaperones and defenders of localized tissue homeostasis and are befittingly equipped with an extensive spectrum of sensing molecules that vary among tissue macrophages based on signals expected to be present in their respective tissues of residence. These local tissue-derived polarization cues offer a plausible explanation for the heterogeneity of tissue macrophages (3). As an example of a tissue-specific macrophage population that shifts between phenotypes in injured skeletal muscle tissue, Sciorati and his colleagues refer to the dynamic macrophage phenotype transition as being “causally connected to and representing limiting steps for muscle healing” (33). It becomes increasingly clear that macrophage phenotypic transition both regulates and reflects the progression of the inflammatory response from initiation to healing. This offers room for speculation as to whether this process can be actively manipulated. Fascinatingly, macrophages can regulate their own phenotype and shape immune responses *via* mechanisms that include, but are not limited to resolution feedback circuits (34) and release of growth factors and microvesicles following phagocytosis (35). For example, annexin A1 (AnxA1, also known as lipocortin-1) an endogenous glucocorticoid-regulated protein that functions to restore homeostasis by counter-regulating inflammatory events (36, 37), is an anti-inflammatory mediator as well as an inducer of macrophage polarization towards the pro-resolving phenotype (38). Additional pro-resolving agents and macrophage reprogramming enhancers are detailed in **Table 1**.

**Table 1:** Pro-resolving effectors and macrophage reprogramming enhancers.

Molecule	Key Mechanisms on macrophages	Effects on Pathology	Reference
*Annexin A1*	↑ monocyte recruitment ↑ efferocytosis ↓ production of proinflammatory cytokines ↑ macrophage reprogramming toward a resolving phenotype	↑ tissue homeostasis ↓ inflammation	(34, 35)
*Peptide Ac 2-26 (from* *Annexin A1)*	↑ Macrophage accumulation	↓ inflammation	(36)
*LXA_4_, 15-epi-LXA_4_,*	↑ efferocytosis	↑ resolution ↑ bacterial clearance ↑ survival	(37–40)
*Resolvin D1*	↑ efferocytosis ↓ TLR-mediated activation of macrophages ↑ M2 polarization ↑ efferocytosis ↑ autophagy ↑ heme oxygenase 1 ↓ apoptosis	↓ inflammation ↓ fibrosis ↑ renal function	(32, 41–46)
*17-epi-resolvin D1*	↑ efferocytosis	↑ resolution ↑ bacterial clearance	(40)
*Resolvin D2*	↓ classically activated macrophage ↑ M2 polarization ↑ Heme Oxygenase 1	↓ inflammation ↑ survival	(47–49)
*Resolvin D3*	↑ macrophage reprogramming toward M2 phenotype ↑ Efferocytosis ↑ Phagocytosis	↓ inflammation ↑ resolution	(50–52)
*Resolvin D4*	↓ macrophage recruitment to thrombus ↑ efferocytosis	↓ inflammation ↑ resolution	(53, 54)
*Resolvin D5*	↓ macrophage- derived pro-inflammatory genes	↑ resolution ↑ survival	(55)
*Resolvin E1*	↑ efferocytosis ↑ Heme Oxygenase 1 ↑ anti-inflammatory cytokine production	↓ inflammation ↓ tissue injury ↑ resolution ↑ survival	(32, 39, 41, 56–58)
*Resolvin E2*	↑ anti-inflammatory cytokine production ↑ phagocytosis	↓ inflammation ↑ resolution	(59)
*Maresin 1*	↑ efferocytosis ↑ Heme Oxygenase 1	↓ inflammation Organ protection ↓ tissue hypoxia ↓ edema	(41, 60, 61)
*Protectin D1*	↓ TLR-mediated activation of macrophages ↑ efferocytosis ↑ Heme Oxygenase 1	↑ renal function ↓ fibrosis ↑ resolution	(39, 41, 42, 62)
*Protein S/GAS6*	↑ efferocytosis	↑ resolution	(63)
*DEL-1*	↑ efferocytosis	↑ resolution ↓ inflammation	(64)
*Galectin-1*	↑ efferocytosis ↑ IFN-β induction ↑ macrophage reprogramming toward pro-resolving phenotype	↑ resolution ↓ inflammation	(65–67)
*Interferon-β*	↑ efferocytosis ↑ macrophage reprogramming toward pro-resolving phenotype ↑ transcriptomic transition that resists tissue fibrosis and oxidative damage	↓ fibrosis ↑ resolution	(29, 68)
*Elafin (PI3)*	↑ macrophage reprogramming toward M2 phenotype ↑ efferocytosis ↑ Annexin A1	↑ resolution	(69)
*TGF-β*	↑ macrophage recruitment regulation ↑ IFN-β induction ↑ efferocytosis	↑ resolution ↑ wound healing	(21, 29, 45, 70, 71)
*IL-10*	↑ macrophage reprogramming toward M2 phenotype	↓ inflammation ↑ homeostasis	(21, 72)
*Arginine*	↑ efferocytosis	↑ resolution	(73)
*IL-4 or IL-13 +* *apoptotic cells*	↑ induction of classical anti- inflammatory and tissue repair genes	↓ inflammation ↑ wound healing	(74)
*IGF1*	↑ macrophage reprogramming toward M2 phenotype	↑ resolution ↓ inflammation	(75)
*Lactate*	↑ macrophage reprogramming toward M2 phenotype ↑ efferocytosis	↓ inflammation	(76, 77)
*Plasminogen/plasmin*	↑ annexin A1 induction ↑ macrophage reprogramming toward M2 phenotype ↑ efferocytosis	↑ resolution ↓ inflammation	(78, 79)

↓ Decrease. ↑ Increase.

## Efferocytosis: Mechanisms and Outcomes

### Role of Apoptosis in Homeostasis and Pathology

Pro-inflammatory macrophages phagocytose damaged cells (which have undergone necrosis, or uncontrolled cell death) or pathogens to induce inflammation. Pro-resolving macrophages, however, are instrumental in anti-inflammatory responses, i.e. the phagocytosis of cellular debris or apoptotic cells and the production of anti-inflammatory/suppressive cytokines and pro-resolving lipid mediators (39, 40). Apoptosis refers to a form of programmed cell death (PCD) that promotes elimination of excess cells generated during development (39, 41, 42), aging cells that have reached the end of their lifespan (39, 41, 43), cells associated with tissue remodeling (33, 39) and cells that have been damaged due to a plethora of microenvironmental factors (33, 39, 44). Apoptosis is mediated *via* the extrinsic or intrinsic pathways, also termed type I and type II apoptosis, respectively (45–47). The pathway is activated by the ligation of “death receptors” on the cell surface, with ligands such as Fas or TNF–α (45, 48–50). The intrinsic pathway is mitochondria-dependent and associated with cytotoxic or oxidative cellular stress, cytokine deprivation, or damage to the genome (48, 51). Both pathways result in activation of the caspase cascade, leading to activation of effector caspases, which induces intracellular substrate degradation, nuclear condensation, cell membrane blebbing, cell shrinkage, and outer membrane cellular changes (47, 48, 52). Failure of immediate removal of apoptotic cells may lead to post-apoptotic cytolysis, or secondary necrosis (39). Necrosis results in the release of cell constituents that generate pro-inflammatory signals. Therefore, removal of apoptotic cells *via* efferocytosis, the term which refers to the phagocytic engulfment of apoptotic cellular corpses by macrophages and their consequent production of pro-resolving signals, is critical for maintaining homeostatic turnover (53, 54).

### Neutrophil Apoptosis

Human circulating neutrophils have a short half-life (55, 56). Mature neutrophils undergo constitutive apoptosis that renders them unresponsive to exogeneous stimuli and facilitates their removal by macrophages *via* efferocytosis (57–60). At sites of inflammation, neutrophil survival and apoptosis is profoundly influenced by opposing cues from the inflammatory microenvironment, including PAMPs, DAMPs and environmental factors (61, 62). Extended neutrophil lifespan through delayed apoptosis is associated with increased disease severity or poor outcome, as observed in patients with asthma (63), sepsis (64), or acute coronary artery disease (65). Preclinical studies showed that delaying neutrophil apoptosis can amplify and perpetuate the inflammatory response (66, 67). For example, myeloperoxidase released from neutrophils triggers a feedforward loop to delay neutrophils apoptosis and consequently resolution of inflammation (66). Conversely, treatment with cyclin-dependent kinase inhibitors (68), 15-epi-lipoxin A_4_ (69) or IFN-β (70) efficiently counter pro-survival cues, redirects neutrophils to apoptosis and facilitates efferocytosis. Typically, phagocytosis of bacteria also accelerates neutrophil apoptosis (62). Consistently, impaired phagocytosis is associated with reduced capacity to clear bacteria and prolonged neutrophil survival. For example, TLR9 ligation with bacterial or mitochondrial DNA leads to degranulation of primary granules, and neutrophil elastase and proteinase 3-mediated cleavage of complement C5a receptor, which together with Mac-1 (or complement receptor 3/CD11bCD18) mediates phagocytosis, resulting in impaired phagocytosis both *in vitro* and in mice (71). Aspirin triggered 15-epi-LXA_4_ and 17-epi-RvD1 signaling through the formyl peptide receptor 2/lipoxin A receptor was shown to counter TLR9 signaling, to restore phagocytosis and accelerate neutrophil apoptosis and resolution of bacterial infections (71). Furthermore, RvD1, acting through the LTB_4_ receptor BLT1 (72), and RvD5, signaling through GPR32 (73) can enhance phagocytosis by naïve neutrophils to promote the resolution of bacterial infections. Of note, neutrophils carrying certain types of bacteria (e.g. toxoplasma or leishmania) that they cannot destroy, may serve as “Trojan horses” to disseminate the infection on macrophage engulfment (74, 75).

Facilitating neutrophil apoptosis is critical to minimizing damage to the surrounding tissue and is an important mechanism to assure removal of emigrated neutrophils from the site of infection or tissue damage. Uptake of apoptotic neutrophils by macrophages influence their function and phenotype, which in turn could modulate the fate of neutrophils (61, 62). For example, IFN-β secreted by macrophages satiated with apoptotic neutrophils mediates both feedback and bidirectional crosstalk between non-phagocytic macrophages, phagocytic macrophages and neutrophils to enhance resolution of inflammation (70).

### Efferocytosis

Efferocytosis is a key process in the resolution of inflammation, which requires selective recognition. The immune system is constantly screening the body for pathogenic invaders by way of ensuring host defense. Therefore, it is of utmost importance to differentiate between phagocytosing in order to trigger acute inflammation or to maintain homeostasis, a process which is responsible for removing over 10^11^ cells daily in the healthy adult without inducing inflammation (76). This dichotomy in “decision making” is resolved by “find me/eat me” signals released by apoptotic cells, which recruit phagocytes to their vicinity and identifies them as efferocytic targets (77, 78). The “find me” signals include the nucleotides ATP and UTP, and the lipid mediators lysophosphatidylcholine (LPC) and sphingosine 1-phosphate (S1P). Proteins, such as the chemokine CX_3_CL1, ribosomal protein S19 (RPS19, in its dimeric form), endothelial monocyte activating polypeptide II (EMAP II/AIMP1), and tyrosyl tRNA synthetase (TyrRS) are also released from apoptotic cells and exert chemoattractant properties. Notably, all these effectors bind to GPCRs on phagocytes and consequently attract them to the apoptotic cell designated for engulfment. In addition to governing the directional movement of macrophages, some of these mediators also regulate macrophage activation and/or polarization, highlighting the ability of apoptotic cells to influence phagocyte activity before and/or after efferocytosis. The most-studied apoptotic cell “eat me” signal is a component of the cell plasma membrane, phosphatidylserine, which is found on the inner lipid bilayer leaflet in healthy cells and becomes exposed on the cell surface when the cell undergoes apoptosis (due to altered functionality of scramblases and flippases) (54). It is plausible that this plasma membrane alteration physically enables phagocytosis independent of chemical signaling (45). Indeed, insertion of phosphatidylserine into erythrocytes allows macrophages to recognize and engulf red blood cells, identifying phosphatidylserine as a central “eat me” signal for phagocytes (78). Furthermore, healthy cells also express “don’t eat me” signals, for example CD47, which repels phagocytes (39). CD47 engagement of SIRPα on macrophages was identified as an important phagocytosis-inhibiting signal (54). In addition to the “find me” signals just described, mediators, such as lactoferrin, released from apoptotic cells were identified as possible mediators that limit the recruitment of inflammatory leukocytes, including neutrophils and eosinophils, thereby enhancing inflammation resolution (45). Perry et al. have identified a solute carrier 12A2 (SLC12A2)-dependent chloride-sensing pathway, which regulates chemotaxis of phagocytes to apoptotic cells as well as their subsequent phenotype-related anti-inflammatory response, providing a mechanistic link between macrophage emigration and efferocytosis (40). This differentiation is considered an efferocytosis-induced physiological “switch” from macrophage-mediated inflammation to macrophage-mediated resolution. This phenotype conversion can be identified by engulfment-dependent production of anti-inflammatory mediators, adenosine, and prostaglandin E_2_ (79). Intriguingly, post-efferocytotic macrophages, termed “satiated/CD11b^low^ macrophages”, were suggested to promote “changing of the guards” by upregulating the chemokine receptor CXCR4 on macrophages following apoptotic engulfment (37, 77, 80). CD11b down-regulation and CXCR4 expression are associated with decreased phagocytic capacity (a characteristic feature of the M2-like phenotype) (37) as well as with macrophage egression *via* the draining lymph nodes to remote organs in response to CXCL12, the CXCR4 ligand (81). Considering the phenomenon of satiated macrophages upregulating CXCR4 expression (50), a likely scenario emerges in which macrophages that lost their capability for efferocytosis make way for macrophages with intact phagocytic capacity. CXCL12/CXCR4-dependent migration of satiated macrophages is also operational for monocyte recruitment (34, 82). This would likely ensure continued clearance of cell corpses during the resolution of inflammation, as has been proposed to occur in the presence of the pro-resolving lipid mediator resolvin E1 (RvE1), dexamethasone, or the pro-resolving cytokine IFN-β (34, 36). Thus, removal of satiated macrophages from the front lines represents a supplementary mechanism to the primary outcomes of efferocytosis, namely: termination of inflammation, activation of pro-resolving pathways, and promotion of self-tolerance (83). Furthermore, efferocytosis also leads to downregulation of proinflammatory cytokine expression, inhibition of inducible nitric oxide synthase (iNOS), and enhanced generation of angiogenic growth factors (83–86).

A key route by which pro-resolving pathways are activated include the production of specialized pro-resolving mediators (SPMs) (87), as well as the upregulation of genes involved in the generation of T_reg_ cells, which were shown to enhance macrophage efferocytosis through the IL-13-IL-10 axis (88). Bonnefoy and his colleagues refer to the secreted anti-inflammatory and pro-resolving mediators collectively recovered from efferocytic macrophages as SuperMApo; *Sup*ernatant of *M*acrophages eliminating *Apo*ptotic cells (89). These mediators are adept at limiting autoimmunity in various organs primarily through TGF-β signaling (90, 91). Inefficient priming of CD4^+^ T cells and the diversion of autoantigens towards recycling endosomes and away from the MHC class II-loading compartment promote self-tolerance. Together, these two processes result in the evasion of T cell responses in favor of apoptotic fragment engulfment (83). Even if this mechanism was impaired, peptides originated from apoptotic cells would still be rendered unsuitable for MHC class II presentation since phagosomes in pro-resolving macrophages are more prone to acidification than those in pro-inflammatory macrophages, resulting in a more efficient proteolysis of apoptotic cell-derived-peptides (92).

### Autoimmunity and Autoinflammation

The ability of resolution-phase macrophages to prevent immune responses and their proficient antigen-presentation evasion would imply that failure in efferocytosis promotes the development of autoimmunity (83). Inefficient apoptotic cell engulfment by macrophages, which can result from an uneven ratio of macrophage to apoptotic cell populations has been suggested as a potential etiology of autoimmunity. Thus, upon inefficient (or lack of) engulfment, apoptotic cells may undergo secondary necrosis, or post-apoptotic cytolysis, which results in the expression of alarmins, a diverse set of endogenous molecules that are released upon unregulated cell death and/or degranulation or damage-associated molecular patterns (DAMPS) (93). When phagocytic cells are unable to distinguish between damage and pathogen-associated molecular patterns, or DAMPS and PAMPS, respectively, autoreactive inflammation can occur as a result of DAMPS activating the inflammatory process generally induced by PAMPS; activation of complement and somatic hypermutation-induced in autoreactive B-cells (46, 94). Moreover, undigested necrotic cell DNA can promote the formation of large immune complexes, which trigger the production of high levels of inflammatory cytokines, such as TNF-α and type I interferon, thereby promoting autoimmune diseases (48). Several studies have shown that apoptotic cell-stimulated interleukin-10 (IL-10), a major immunoregulatory cytokine, plays a crucial role in the prevention of autoimmunity (95, 96). Originally perceived as a Th2 cytokine that suppresses cytokine production by Th1 cells, IL-10 plays a central role in restricting inflammatory responses *in vivo*. Macrophages produce large amounts of IL-10 following cell-to-cell contact with apoptotic cells, but not phagocytosis (95). This underscores the ability of organisms to anticipate potential failure (i.e. autoimmunity that can evolve from flawed efferocytosis) and prepare itself *via* production of immunoregulators, accordingly (95). In addition to IL-10 regulation, compensatory mechanisms such as the CD24-Siglec G pathway can protect the host from apoptotic-debris-induced-autoimmunity by discriminating between DAMPS and PAMPS (46, 94).

## Pro-Resolving Effectors Can Limit Infection-Associated Cytokine Storm

### Defining the Cytokine Storm

Autoimmunity or a cytokine storm (also known as cytokine release syndrome (CRS), or Hypercytokinaemia) are two potential disastrous outcomes of an uncontrolled immune response. Cytokine storm is currently defined as markedly elevated levels of inflammatory cytokines in body fluids, resulting in vascular damage, immunopathology, and deteriorating clinical outcomes (97). Under ideal conditions, the defensive inflammatory response is proportional to the severity of infection. The balance is achieved by regulatory mechanisms, such as resolution of inflammation, which limits hyperreactivity in space and time. Failure to enter the phase of resolution and repair may lead to further increases in levels of inflammatory cytokines, which characterize the Cytokine Storm. These cytokines include IL-1, IL-2, IL-6, GM-CSF, IFN-γ, and TNF-α and their transcription is predominantly driven by NF-κB. These cytokines interact with the complement and coagulation systems to induce disseminated intravascular coagulation (DIC), acute respiratory distress syndrome (ARDS), hemophagocytic lymphohistiocytosis (HLH), and ultimately multiorgan failure (97, 98). This phenomenon is induced by enhanced innate recognition *via* elevated production of IL-2, IFN-γ, and TNF-α, highlighting that protective immunity can become deleterious when it is dysregulated or when T cells escape many of the cell-extrinsic checkpoints (97, 99). Genetic defects, leading to disproportionate inflammasome activation and sustained IL-1 production by macrophages, may also contribute to the development of the cytokine storm (97). Moreover, the generation of reactive oxygen/nitrogen species (ROS/RNS) in the lungs as well as in the mitochondria provokes inflammatory responses. Oxidative and/or nitrosative stress activates signaling pathways, including NF-κB, which leads to the upregulation of proinflammatory cytokines and chemokines, thereby contributing to the development of the cytokine storm (100, 101).

### Resolution Cues

Therapeutic attempts to limit the cytokine storm can harness the reprogramming of M1 macrophages to the M2 phenotype. In addition to apoptotic cell-induced production of pro-resolving cytokines, like TGF-β and IL-10, reprogramming is accomplished *via* resolution cues, which operate in autocrine/paracrine manners, and result in downregulation of pro-inflammatory mediators (also termed immune-silencing) (95, 96, 102). These resolution cues were originally shown to be mediated by prostaglandin E_2_ (PGE_2_) and platelet activating factor (PAF) (103), with more recent studies elucidating their intricate and potentially opposing actions (104–107), underscoring the dynamism of homeostatic maintenance. Key pro-resolving effector molecules include proteins, peptides, SPMs and gaseous mediators. SPMs are derived from arachidonic acid and omega-3-polyunsaturated fatty acids including lipoxins, resolvins, protectins, and maresins (108, 109). SPMs do not compromise host defense, while they actively promote resolution and the return to homeostasis (37, 110–112). Galectins, annexin A1 (AnxA1) and peptides derived from AnxA1 serve various roles in counter-regulating inflammatory events (38, 102).

With regard to resolution effector receptors, or immune-silencing at the phagocyte receptor expression level, stimulation of various phagocytic adenosine receptors was found to inhibit inflammation by diminishing leukocyte recruitment to the site of inflammation through inhibition of both selectin- and integrin-mediated adhesive events (113). Furthermore, five separate GPCRs; ALX, DRV1, ERV, BLT1 AND DRV2 (87), which selectively bind individual SPMs, are upregulated during the acute inflammatory reaction (114), counter-regulate proinflammatory pathways (115, 116) and stimulate protective gene expression (117). These processes are also driven by ligands on the surface of apoptotic cells, such as ACKR2-bound CCL5 (118, 119), “eat me” signals (or the absence of “do not eat me” signals), cognate receptors on the phagocytes, and/or bridge molecules in the environment (85, 120).

CCR5 expression on late apoptotic human neutrophils was found to modulate macrophage numbers and phenotype reprogramming by clearing its inflammatory ligands during the resolution of peritonitis (121). Accordingly, SPMs were shown to upregulate CCR5 expression on apoptotic neutrophils, parallel with downregulation of pro-inflammatory cues. Thus, CCR5^+^ apoptotic neutrophils may act as ‘terminators’ of chemokine signaling during the resolution of inflammation (121, 122). Resolution mediators also include effector cytokines, such as the macrophage-derived IFN-β. IFN-β is a type I interferon that regulates anti-viral and anti-bacterial immune responses (123). Resolution phase macrophages that have lost their phagocytic capacity express a distinct IFN-β-related gene signature in mice compared to their phagocytic ancestors (34). Correspondingly, elevated levels of IFN-β have been reported in resolution-phase peritoneal and broncho-alveolar exudates in mice, and IFN-β, in turn, enhances bacterial clearance, neutrophil apoptosis, efferocytosis, and consequent macrophage reprogramming (34). An additional master-regulator of resolving inflammation is the pro-apoptotic protein ARTS. ARTS is a mitochondrial protein that limits neutrophil survival and possesses pro-resolving actions similar to those of SPMs and cyclin-dependent kinases (68, 124), namely promoting neutrophil apoptosis, efferocytosis, and the reprogramming of macrophages to the pro-resolving phenotype (124).

### Resolution Pathologies

Whereas the above-mentioned mechanisms represent the ideal outcome of inflammation, in the “real-life” immune battlefield, disturbances can and do occur, which may underlie diseases. Given the plethora of molecules, signaling pathways, and cascades involved in the immune response, the potential disturbances and resulting pathologies are numerous. We will discuss the molecular mechanisms underlying multiple pathological conditions that are associated with incomplete resolution of inflammation, fibrosis, Macrophage Activation Syndrome (MAS), and autoimmunity.

In the case of abnormal resolution-phase tissue repair involving the deficient generation of pro-resolving macrophages and resulting in uncontrolled production of inflammatory mediators, growth factors and ECM components, pathological fibrosis may occur when endothelial cells, fibroblasts, and stem or tissue progenitor cells collectively reinforce a state of persistent injury and/or exaggerated repair (125). This pathological fibrotic state begins with the recruitment of fibroblasts to the inflamed tissue, and consequently their differentiation into extracellular matrix (ECM)-producing myofibroblasts that assist in repairing the damaged tissue. This innocent, commonplace process may take a sinister turn in the prolonged presence of pro-fibrotic mediators that elicit disproportionate, and long-lasting recruitment, and activation of myofibroblasts (126). Pathological fibrosis is characterized by the excessive deposition of ECM, including type I and III collagens, fibronectin, and laminin (127). TGF-β induces upregulation of collagen synthesis, epithelial-mesenchymal transition (EMT), and myofibroblast trans-differentiation; consistent with fibroblast activation. Curiously, inhibition of TGF-β1 requires mediators that can be produced by macrophages following apoptotic cell uptake (i.e. when they became satiated), implying an antifibrotic role for these macrophages under certain settings (127, 128). Some myofibroblasts can remain plastic and naturally convert to different cell types, representing a renewal potential that depends on their origin (126, 129). These findings challenge the widely held notion that fibrosis and wound healing are opposing processes. Furthermore, studies on hair follicle neogenesis support the concept that the mechanisms of scarring and normal tissue remodeling are not distant from one another, and that wound repair can be redirected to promote regeneration following injury by modifying tissue signals (130).

Synonymous with secondary hemophagocytic lymphohistiocytosis, MAS is an umbrella term for systemic hyper-inflammation that can potentially be life-threatening. Markedly elevated levels of the acute-phase reactant C-reactive protein, IL-6, IL-7, TNF-α and hyperferritinemia (excess of the iron storage protein ferritin), are the key diagnostic criteria for MAS (131). Concentrations of the α-chain of the IL-2 receptor (132) are also increased. The pathogenesis of MAS is thought to involve a defect in lymphocyte cytolytic activity, which results from a pro-inflammatory environment. Decreased cytolytic activity likely amplifies the pro-inflammatory cytokine cascade, resulting in a cytokine storm (133). A plausible etiological cause for MAS is macrophage phagocytosis of red blood cells, which, although critical for the clearance of infected cells, results in continuous production of IFN-γ and TNF-α. The sustained pro-inflammatory environment and the resulting decreased cytolytic activity enhance the probability of MAS occurrence (97).

Autoimmunity refers to the immune system targeting host cells. Failed efferocytosis, or inefficient apoptotic cell engulfment by macrophages, can result in autoimmunity following secondary necrosis, and release of DAMPs. DAMPs can, in turn, trigger inflammation through activation of their cognate pattern recognizing receptors (PRR) that cannot differentiate between PAMPs and DAMPs. The generation and deposition of immune complexes, and activation of the inflammasome, contribute to development of autoimmune diseases (46, 48, 83, 94).

## Implications for COVID-19

### Harnessing the Resolution of Inflammation to Limit COVID-19

The emergence of COVID-19 as a worldwide pandemic and the appearance of associated pulmonary pathology similar to that of lung fibrosis, coupled with deficiencies in pro-resolving mediators, raise the possibility of utilizing satiated macrophages and/or their products for the treatment of this infection. Curiously, SARS-CoV-2 infection remains asymptomatic or causes mild flu-like symptoms in 80% of people, whereas others develop overt pneumonia that is associated with high mortality (134). COVID-19 offers a fascinating window into the mechanisms by which the immune state of the host may affect disease progression. Viewing COVID-19 from the macrophage phenotype continuum to cytokine expression perspective, it appears that the host immunological predisposition and potential deficiencies may be critical for determining mild or severe disease manifestations. Marked changes in immune cell compositions, phenotypes, and cross-talk were identified in SARS-CoV-2-infected individuals, and clear distinguishing features were observed between mild and severe cases (70, 98, 123, 132, 134, 135).

To comprehend the difference between the onset of mild and severe disease, one may consider COVID-19 as a two-phase disease, in which virus pathology dominates the early phase and immunopathology dominates the latter. The early phase of infection involves SARS-CoV-2 binding to angiotensin-converting enzyme 2 (ACE2) (93) and the activation of the type I interferon response, which inhibits viral replication (98, 136). In most infected people, this phase is characterized by mild or moderate symptoms (which arise from virus-associated tissue damage and antiviral activity of the adaptive immune system) and is successfully resolved through a regulated antiviral immune response (93). The second phase develops only when the immune response becomes dysregulated, with release of alarmins and DAMPS, coupled with the host’s inability to resolve the viral infection (98). At this point, the looming cytokine storm breaks loose, forming a destructive feedforward loop, in which the inflammatory reaction damages tissues, and the lungs in particular, which then triggers further inflammation (93). The lethal trajectory of ARDS, sepsis-like stage, and ultimately organ failure is initiated if the hyperinflammatory feedforward loop is not restrained (93, 97).

Insofar as the early phase of COVID-19 has now become a manageable condition with commercially-available vaccines, current major challenges involve the management of the second phase of the disease, namely the secondary complications in different organs resulting from immunopathology. One such organ is the brain. There are several case reports documenting COVID-induced-demyelination of both peripheral and central nervous systems (137, 138). Demyelination underlies many COVID-19-associated CNS pathologies, including but not limited to encephalitis, acute disseminated encephalomyelitis, meningitis, ischemic and hemorrhagic stroke, venous sinus thrombosis, endothelialitis, anosmia, hyposmia, Parkinsonism, Alzheimer’s diseases and psychiatric symptoms (139, 140). These CNS complications have been attributed to a variety of mechanisms, including virus-induced hyperinflammatory and hypercoagulable states, and post-infectious immune mediated processes. However, as numerous neuropathologies can denote either a direct viral invasion of the CNS, virus-induced hyperinflammatory and hypercoagulable states, or postinfectious immune-mediated processes which may develop as a mere sequel of hypoxia affecting the CNS, from which direct causality cannot be readily inferred (139, 141). Nonetheless, from accumulating evidence indicates that SARS-CoV-2 and several proinflammatory cytokines, including IL-1β, IL-2, IL-4, IL-6, IL-8, IL-10, TNF-α, and IFN-γ, can cross the blood–brain barrier, suggesting a highly probable para- or post- infectious immune-mediated etiology of COVID-19-associated CNS injuries (142, 143). A plethora of mechanisms have been identified *via* which viruses can cross into the CNS, including the transmigration of macrophages or T cells carrying viral particles (144–146). Accumulation of diverse subsets of lymphocytes and inflammatory mediators in the CNS provides an ideal setting for a perfectly orchestrated cytokine storm, as well as an opportunity to explore the role of macrophages cross-talk with the adaptive immune system.

Multiple sclerosis (MS) represents a useful model for understanding COVID-19-associated neuropathology, for it is an immune-mediated demyelinating disease characterized by the accumulation of immune cells in the CNS (147). Its pathogenesis, which evolved through analogy with experimental autoimmune encephalomyelitis (EAE), involves CNS-infiltrating myelin-specific Th cells that induce monocyte conversion to an M1-like phenotype *via* pro-inflammatory mediators, such as GM-CSF. GM-CSF, in turn, aggravates CNS inflammation, and ultimately leads to myelin damage and neuronal loss (148–152). Amplification of this autoimmune neuroinflammation by a feedback loop between monocytes and Th cells can be attenuated by IFN-β therapy, which results in indirect suppression of GM-CSF production by Th cells *via* increasing IL-10 expression by monocytes. This phenomenon underscores the critical role of monocyte and macrophage phenotype conversion in immune regulation (151). Furthermore, in a study on how morphology and function of both neutrophils and microglia affect the inflamed brain, a crosstalk with neutrophils extravasating to the brain parenchyma has been postulated to serve as an additional primer of macrophage-induced neuroinflammation (153). Thus, recruitment of immune cells into the CNS allows for crosstalk between distinct cell types, leading to consequent modulation of the inflammatory response.

### Distinguishing Features Between Mild and Severe Cases

Studies on bronchoalveolar lavage (BAL) fluid cells revealed unaffected monocyte/macrophage antiviral and phagocytic functions in patients with mild COVID-19 (134). In critically ill patients, these monocytic cells activate excessive inflammation in response to sensing PAMPs and DAMPs, which may further be exacerbated by the reduction in monocyte-originated macrophages whose role is to phagocytose the ensuing debris (134, 154, 155). An additional signal to trigger hyperinflammation underlying severe illness is the release of neutrophil extracellular traps (NET) and NET-stimulated coagulopathy (156). NETosis is likely initiated by both direct (through angiotensin-converting enzyme 2, serine protease, virus replication, and PAD-4 (157)) and indirect (through activated platelets and COVID-19-triggered cytokines and chemokines which stimulate NETosis (158)) mechanisms. This results in enhanced coagulation with decreases in lymphocyte counts, an abundance of inflammatory myeloid cells, endothelial cell damage, thrombus formation, and ensuing fibrosis, all of which is associated with a poor COVID-19 prognosis (97, 134, 141, 159). The success of treatment with cytokine receptor antagonists (160) and cytokine neutralization (161) in patients with severe COVID-19 lends further support to the notion of dysregulated inflammatory response being the distinguishing a feature between mild and severe cases (135).

Although it is of utmost importance to understand the pathophysiology underlying severe COVID-19, from the clinical point of view, it will be important to identify differences in the mechanisms underlying mild versus severe disease in order to develop more efficient therapies. While no consensus has emerged to date as to the cause of the deleterious immune response arising from a system intended to be protective, several possibilities should be considered. Mangalmurti and Hunter argue that the key distinguishing factor underlying COVID-19 pathogenesis seems to be preexisting conditions that affect vascular health, including diabetes, hypertension, and other cardiovascular diseases. These comorbidities may decrease resilience and lower host ability to tolerate systemically released cytokines (97). An alternative explanation is a distinct phenotype of an impaired IFN type I response in patients with severe COVID-19. In these patients, viral clearance is disrupted, generating a welcoming environment for hyperinflammation (98, 123). However, other studies detected a robust type I interferon response in association with severe COVID-19 (70). We propose that these apparently conflicting observations can be explained by the two-phase approach toward COVID-19. Thus, deficiency in type I IFN response can dramatically inhibit viral clearance in the first phase where protective inflammation is crucial, whereas excessive production of type I IFN during the second, hyper-inflammatory phase has the potential to hamper the host’s attempt to activate anti-inflammatory and pro-resolving processes.

### Calming the Storm

When it comes to calming a cytokine storm and restoring dysregulated immunity, both inhibition of proinflammatory mechanisms as well as activation of anti-inflammatory processes are of critical consequence. The findings that elevated levels of both IL-6 and IL-10 are predictive of disease severity supports this notion, given that IL-6 is key proinflammatory cytokine, whereas IL-10 is a prototypical anti-inflammatory cytokine whose presence may suggest a protective role in disease progression (143). Timeliness of initiating therapy appears to be critical, for immunosuppression during the antiviral phase of the COVID-19 can have detrimental effects, whereas it could potentially break the propagating feedforward loop and even initiate resolution during the hyperinflammatory stage.

**Figure 2:**
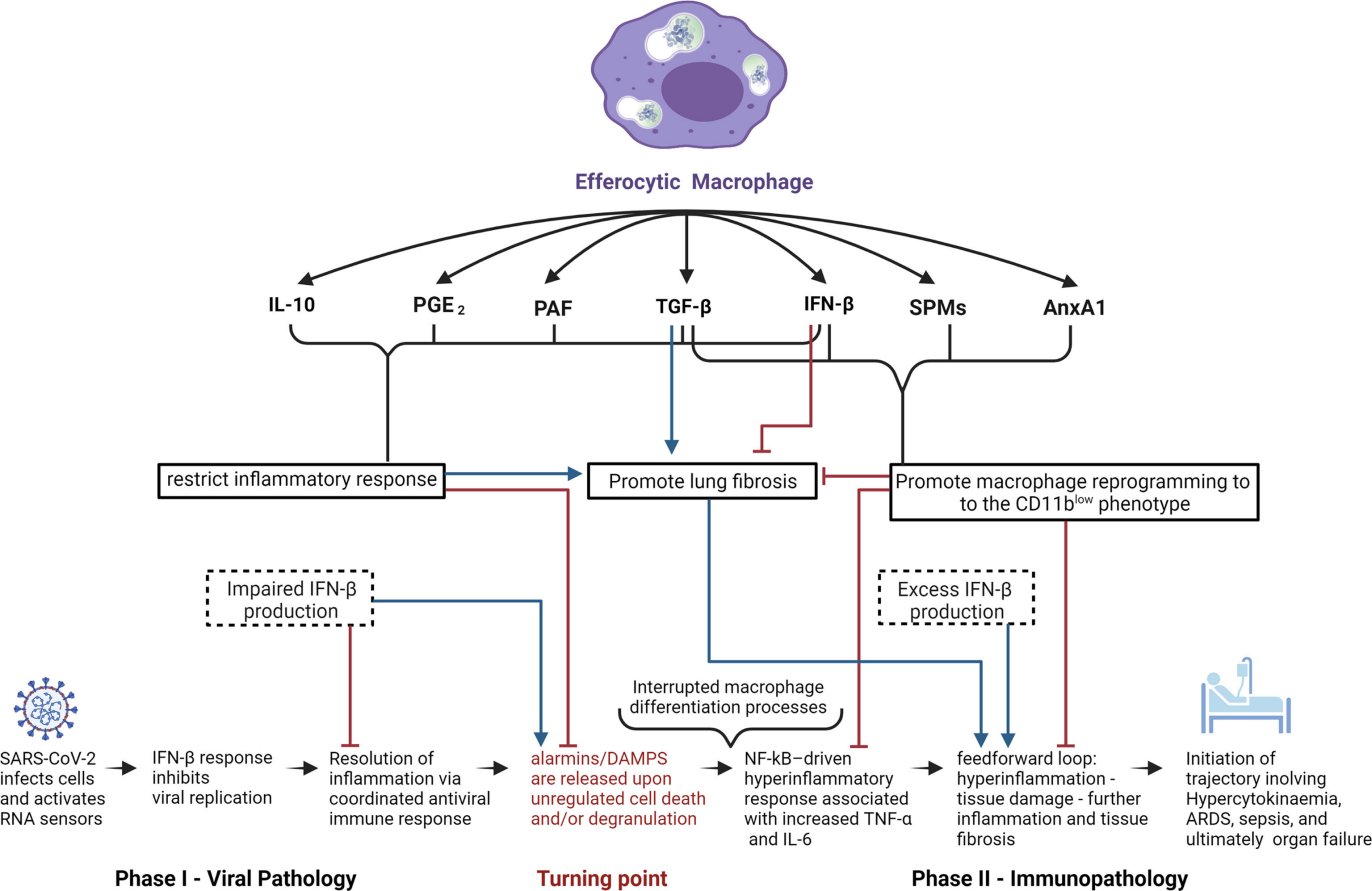
Potential role of efferocytic macrophages in COVID-19 pathogenesis. Schematic representation of potential mechanisms by which efferocytic macrophage-produced mediators could affect COVID-19 pathogenesis. Anti-inflammatory effectors IL-10, PGE_2_, and PAF all restrict the inflammatory response which, while crucial for inhibiting the progression of viral infection and immunopathology, have the potential to promote fibrosis. SPMs and AnxA1 can inhibit fibrosis by promoting macrophage reprogramming towards the CD11b^low^ phenotype, parallel with inhibition of the feedforward hyperinflammatory loop. The cytokines TGF-β and IFN-β (at low concentrations and when temporally restricted) have the ability to inhibit fibrosis, due to their ability to restrict the inflammatory response as well as to promote macrophage reprogramming to the CD11b^low^ phenotype. On the other hand, chronic exposure to high doses of TGF-β or IFN-β may result in pathologic fibrosis or autoinflammatory disorders. In the light of IFN-β’s anti-viral and anti-inflammatory properties, IFN-β deficiency during phase I of COVID-19 infection and excess IFN-β production during phase II can have deleterious consequences in disease progression and inflammation resolution.

With regard to activation of anti-inflammatory processes, it is important to note the innate inclination of the immune system to elevate the levels of inhibitory cytokines, including but not limited to IL-1RA and IL-10 at early stages of infection in order to prevent potential development of hyperinflammation (162). Moreover, satiated or CD11b^low^ macrophages are perceived to be central effectors in the restoration of dysregulated immunity given their capacity to regulate and induce expression of anti-inflammatory effectors (37) (**Figure 2**). This perspective coupled with the capacity of glucocorticoids to regulate pro-resolving macrophages is supported by the Randomized Evaluation of COVID-19 Therapy (RECOVERY) clinical trial, which reported very promising results with dexamethasone, a synthetic glucocorticoid in patients with severe ARDS (93, 163). Glucocorticoids exert extensive immunomodulating effects *via* cell-specific changes in the transcriptome by binding to intracellular glucocorticoid receptors (GRs) (164). Consequently, GRs translocate to the nucleus to modulate transcription, while simultaneously affecting non-transcriptional processes *via* plasma membrane interactions and intervention with cytoplasmic signaling cascades and mitochondrial translocation (93). This wide array of physiological effects results in activation of a multitude of pro-resolution and anti-inflammatory mechanisms, including downregulation of genes associated with TCR, BCR, and TLR7 signaling (93, 164–166), significant increases in apoptotic neutrophil phagocytosis by macrophages (167) and monocyte-derived iDCs (168), inhibition of DC antigen presentation and expression of costimulatory molecules (93), upregulation of the anti-inflammatory cytokine IL-10 (93, 169), and the inhibition of production of pro-inflammatory cytokines (*i.e.* IL-1, TNF-α, and IL-6), chemokines, and other soluble mediators (leukotrienes, and histamines) (93, 168). The relevance of these anti-inflammatory properties to COVID-19-induced immunopathologies is further highlighted by the accumulating evidence showing association of markedly elevated levels of IL-6 and other pro-inflammatory cytokines in patients with severe COVID-19 clinical outcomes (170).

AnxA1 and its mimetic peptides, including peptide Ac2-26, have been shown to drive the resolution of inflammation not only as facilitators of efferocytosis, but also by inducing macrophage reprogramming to the CD11b^low^ phenotype and modulating subsequent monocyte recruitment. A notable pro-resolving effect observed with dexamethasone, resolvin D1 and resolvin E1 is the decreased threshold of engulfment-related-events required for inflammatory macrophages to undergo immune silencing and differentiate into the CD11b^low^ phenotype (37). This enhances immune-silencing events and thereby increases the rate of apoptotic cell clearance, providing a mechanism to interrupt the inflammation-damaged tissue-inflammation feedforward loop that maintains cytokine storms (38, 93, 171). It is important to note that glucocorticoids have also been found to drive pro-inflammatory processes (168), highlighting the complexity of their actions in a context, cell type, dose, and timing of exposure-dependent fashion (168).

An alternative approach for calming the cytokine storm is the administration of type I IFN (98). Since hyperinflammation may arise as a consequence of inefficient efferocytosis (which can be the result of a hampered antiviral response and/or reduced IFN-β production (46)), administration of type I IFN can hypothetically prevent hyperinflammation in patients with an impaired ability to generate an antiviral immune response in the initial phase of the disease (98). Indeed, four clinical trials reported a favorable response to early IFN-β use in COVID-19 patients, and other studies are underway to test the timing and clinical efficiencies of IFN-α or IFN-β (70). However, considering the potential danger of propagating lymphocytic immune responses into a long term hyperinflammatory state, appropriate supplementary inhibitors, as well as nuanced dosing and timing of treatment, would be critical to firmly establish this therapy (70).

Regulatory T cells (T_regs_) have also been identified as important coordinators of immune regulation in COVID-19 (172). Thus, COVID-19 patients exhibit an exhausted T cell phenotype as evidenced by the elevated expression of inhibitory immune checkpoints and reduced expression of cytokine and cytolytic molecule genes (173). These findings open new avenues for potential therapeutic interventions aimed to improve antiviral T‐cell responses against SARS‐CoV‐2. These approaches include adoptive T‐cell therapies, T‐cell response-activating vaccines, recombinant cytokines, Th1 activators and Th17 blockers, and immune checkpoint inhibitors used alone or in combination with anti‐inflammatory drugs (173). A plethora of additional repurposed anti-inflammatory therapies for COVID-19 have been proposed, with major emphasis on the critical necessity for vigilance for cardiotoxicity as well as other possible off target effects (163, 174, 175). **Table 2** summarizes multiple immunopathological mechanisms associated with a dysregulated immune system together with potential alternative therapies.

**Table 2:** Dysregulated immunity-associated pathologies and potential corrective therapies.

Dysregulated immune mechanism	Immunopathology	Therapies	References
Viral infection and ensuing replication results in TLR recognition of PAMPs	Pathological NETosis	Heparin-based anticoagulation, dexamethasone, DNases, dipyridamole and complement inhibitors	(158)
Expression of type I (α/β) IFNs and a variety of IFN-stimulated genes and resulting production of inflammatory cytokines and chemokines	Impaired IFN type I response	Administration of type I IFN	(70, 98, 123)
Activation of NK cells and complement	Natural killer cell deficiency	Acyclovir, gancyclovir and related anti-viral drugs	(176)
	Complement deficiencies and dysregulation	Inhibition of contact phase (CP) proteases and inhibition of bradykinin (BK)-mediated effects at the BKR2 on endothelial cells	(177, 178)
Antibody deficiencies	Impaired antimicrobial defense and dysregulated autophagy	mTOR inhibitors	(179, 180)
B and T cell maturation in lymphoid tissues resulting in antigen-specific B and T-cell responses via ligand binding or release of antibodies or cytokines	Hyperactivation of T cells due to evasion of many cell- extrinsic immune checkpoints	Chimeric Antigen Receptor (CAR) Treg- therapy	(99, 172, 181)
Apoptosis of virus-infected cells	Generation of immune complexes and inflammasome activation due to PRR activation by DAMPs/alarmins released upon unregulated cell death and/or degranulation	Selective disruption of TLR2/MyD88 signaling	(182, 183)
Phagocytosis of damaged cells or pathogens induces inflammation	Sustained inflammation and decreased cytolysis due to unbridled activation of cytotoxic T lymphocytes, natural killer (NK) cells, and macrophages	Immune suppressants, etoposide, and allogeneic hematopoietic stem cell transplantation	(184)
	Excessive IFN type I response	Baricitinib (Janus Kinase inhibitor)	(70, 185)
Efferocytosis of apoptotic remnants	Reduced population of macrophages of monocyte origin	Immune-silencing with glucocorticoids	(134, 165, 166)
	Secondary necrosis		
Phagocyte differentiation into pro-resolving phenotype and production of anti-inflammatory and pro-resolving cytokines	Pathological fibrosis resulting from deficient generation of pro-resolving macrophages and persistent production of inflammatory mediators	Macrophage reprogramming	(37)
Upregulation of genes involved in the generation of Treg cells	Autoimmunity resulting from disrupted Th17/Treg balance	Umbilical cord-derived mesenchymal stem cell (UC-MSC) infusion	(186, 187)
Termination of inflammation	Hyperinflammatory feedforward loop	Immune silencing via cytokine receptor antagonists, cytokine neutralization and/or macrophage reprogramming	(37, 134, 161, 166)

On a broader scale, therapies intended to regulate macrophage polarization have been proposed and displayed promising potential. Relevant examples are decursinol angelate, which inhibits M1 polarization *via* modulation of the NF-κB and MAPK signaling pathways (188) and docosahexaenoic acid, which enhances M2 polarization through the p38 MAPK signaling pathway and autophagy (189). Furthermore, infusion of negatively charged, immune-modifying microparticles (IMPs) have been shown to induce suicide in monocytes, thereby preventing their maturation into inflammatory macrophages (190). Taken together, modulation of macrophage phenotype has critical consequences for a healthy, balanced immune system, given the pivotal role resolution effectors play in immune homeostasis. An illustrative scheme on the potential use of resolution effectors for the treatment of COVID-19 pathologies is depicted in **Figure 2**.

## Conclusions

In conclusion, the reprogramming ability of macrophages into efferocytotic and consequently satiated phenotypes emerge as a central driving force for inflammation resolution, without which all immune responses may take a sinister turn in the form of hyperinflammation and ensuing chronicity. The macrophage phenotype continuum provides a framework for phagocyte plasticity in recognizing the host’s actual immune state and consequently responding in an appropriate manner. Understanding the mechanisms by which macrophages travel through this continuum, and how their journey can be manipulated, appears to be the *clavis aurea*, or golden key through which homeostasis can be restored and sustained.

Due to the wide range of SARS-CoV-2 infection phenotypes coupled with the relatively short period of time for research, consistent and definitive correlations of disease stages and precisely defined effectors have not been rigorously identified. However, with these limitations in mind, it is becoming increasingly clear that the state of the host`s immune system may be a critical determining factor for disease progression and eventual outcome. This growing awareness would imply shifting the focus of our research and resources towards the host immune system instead of the perpetrator, SARS-CoV-2. Such efforts, directed at armoring our innate defense system with the necessary manipulations to re-establish dysregulated immune responses and direct them to resolution, will bring us closer to the ultimate goal of homeostatic restoration and prevention of grave immunopathologies, such as ARDS and other long consequences of COVID-19 infection.

## Author Contributions

ES wrote the manuscript draft and designed the figures. Figures were created with BioRender.com. JF and AA discussed the concepts and edited the text. All authors contributed to the article and approved the submitted version.

## Funding

The manuscript was partially funded by the Israeli Science Foundation: grant No. 678/13 and by the Canadian Institutes of Health Research (MOP-102619).

## Conflict of Interest

The authors declare that the research was conducted in the absence of any commercial or financial relationships that could be construed as a potential conflict of interest.

## Publisher’s Note

All claims expressed in this article are solely those of the authors and do not necessarily represent those of their affiliated organizations, or those of the publisher, the editors and the reviewers. Any product that may be evaluated in this article, or claim that may be made by its manufacturer, is not guaranteed or endorsed by the publisher.

## Abbreviations

IFN-γ, interferon-γ; LPS, lipopolysaccharide; GM-SCF, Granulocyte-macrophage colony-stimulating factor; CIITA, class II major histocompatibility complex transactivator; CXCL, chemokine (C-X-C motif) ligand; iNOS, inducible nitric oxide synthase; ROS, Reactive oxygen species; IL, interleukin; Arg, Arginase; SRs, scavenger receptors; MR, mannose receptor; Retnla/Fizz1, Resistin-like molecule alpha1; CCL, Chemokine (C-C motif) ligand; TGF-β, Transforming growth factor-β; Chil3/Ym1, Chitinase-like protein 3; IC, immune complexes; TLR, Toll-like receptor; GC, Glucocorticoid; SPM, Specialized Pro-resolving mediator; PPAR-γ/δ, Peroxisome proliferator-activated receptor-γ/δ; SLAM, signaling lymphocytic activation molecule; MGL1, macrophage galactose-type lectin-1; AnxA1, Annexin A1; PGE_2_, Prostaglandin E_2_; PAF, Platelet Activating Factor; IFN-β, interferon-β; SARS-CoV-2, severe acute respiratory syndrome coronavirus 2; DAMPS, Damage-associated molecular pattern; NF-κB, nuclear factor kappa-light-chain-enhancer of activated B cells; TNF, Tumor necrosis factor; ARDS, Acute respiratory distress syndrome.
